# Genetic aetiologies for childhood speech disorder: novel pathways co-expressed during brain development

**DOI:** 10.1038/s41380-022-01764-8

**Published:** 2022-09-18

**Authors:** Antony Kaspi, Michael S. Hildebrand, Victoria E. Jackson, Ruth Braden, Olivia van Reyk, Tegan Howell, Simone Debono, Mariana Lauretta, Lottie Morison, Matthew J. Coleman, Richard Webster, David Coman, Himanshu Goel, Mathew Wallis, Gabriel Dabscheck, Lilian Downie, Emma K. Baker, Bronwyn Parry-Fielder, Kirrie Ballard, Eva Harrold, Shaun Ziegenfusz, Mark F. Bennett, Erandee Robertson, Longfei Wang, Amber Boys, Simon E. Fisher, David J. Amor, Ingrid E. Scheffer, Melanie Bahlo, Angela T. Morgan

**Affiliations:** 1 Population Health and Immunity Division, The Walter and Eliza Hall Institute of Medical Research, Parkville, VIC 3052 Australia; 2 Faculty of Medicine, Dentistry and Health Sciences, University of Melbourne, Parkville, VIC 3052 Australia; 3 Murdoch Children’s Research Institute, Parkville, VIC 3052 Australia; 4 Neurology Department, The Children’s Hospital at Westmead, Westmead, NSW 2145 Australia; 5 Queensland Children’s Hospital, South Brisbane, QLD 4101 Australia; 6 University of Queensland, St. Lucia, Brisbane, QLD 4067 Australia; 7 Hunter Genetics, John Hunter Hospital, New Lambton Heights, NSW 2305 Australia; 8 School of Medicine and Menzies Institute for Medical Research, University of Tasmania, Hobart, TAS Australia; 9 Tasmanian Clinical Genetics Service, Hobart, TAS Australia; 10 Royal Children’s Hospital, Flemington, Parkville, Melbourne, VIC Australia; 11 University of Sydney, Camperdown, NSW 2006 Australia; 12 Griffith University, Gold Coast, QLD Australia; 13 Victorian Clinical Genetics Services, Parkville, VIC Australia; 14 Language and Genetics Department, Max Planck Institute for Psycholinguistics, 6525 XD Nijmegen, The Netherlands; 15 Donders Institute for Brain, Cognition and Behaviour, Radboud University, 6500 HE Nijmegen, The Netherlands

**Keywords:** Genetics, Molecular biology

## Abstract

Childhood apraxia of speech (CAS), the prototypic severe childhood speech disorder, is characterized by motor programming and planning deficits. Genetic factors make substantive contributions to CAS aetiology, with a monogenic pathogenic variant identified in a third of cases, implicating around 20 single genes to date. Here we aimed to identify molecular causation in 70 unrelated probands ascertained with CAS. We performed trio genome sequencing. Our bioinformatic analysis examined single nucleotide, indel, copy number, structural and short tandem repeat variants. We prioritised appropriate variants arising de novo or inherited that were expected to be damaging based on in silico predictions. We identified high confidence variants in 18/70 (26%) probands, almost doubling the current number of candidate genes for CAS. Three of the 18 variants affected *SETBP1*, *SETD1A* and *DDX3X*, thus confirming their roles in CAS, while the remaining 15 occurred in genes not previously associated with this disorder. Fifteen variants arose de novo and three were inherited. We provide further novel insights into the biology of child speech disorder, highlighting the roles of chromatin organization and gene regulation in CAS, and confirm that genes involved in CAS are co-expressed during brain development. Our findings confirm a diagnostic yield comparable to, or even higher, than other neurodevelopmental disorders with substantial de novo variant burden. Data also support the increasingly recognised overlaps between genes conferring risk for a range of neurodevelopmental disorders. Understanding the aetiological basis of CAS is critical to end the diagnostic odyssey and ensure affected individuals are poised for precision medicine trials.

## Introduction

Childhood apraxia of speech (CAS) is a rare neurodevelopmental disorder, occurring in 0.1% of the population [1]. CAS stems from deficits in speech planning and programming, affecting a child’s ability to sequence sounds and syllables accurately and with correct prosody, resulting in highly unintelligible speech [1, 2]. The first evidence implicating a specific gene in aetiology of CAS was provided in 2001, via a family study revealing that pathogenic variants in *FOXP2* were responsible for the speech disorder [3]. For almost two decades, *FOXP2* was the only gene associated with CAS, in the absence of intellectual disability. Technological advances and reduced costs for DNA analyses have recently enabled efficient genome sequencing and bioinformatic follow-up, paving the way for high throughput discovery of genes involved in CAS. In particular, two independent cohort studies have performed genome-wide sequencing on 52 individuals with CAS [4, 5].

In the first cohort, aetiologic variants were identified in eight of 19 probands ascertained for CAS, yielding a genetic diagnostic rate of 42% with pathogenic variants revealed in: *CHD3*, *SETD1A*, *WDR5*, *KAT6A*, *SETBP1*, *ZFHX4*, *TNRC6B* and *MKL2* [4]. In the second cohort, comprising 33 probands with CAS, nine additional genes were implicated: *CDK13, EBF3, GNAO1, GNB1, DDX3X, MEIS2, POGZ, UPF2* and *ZNF142*. One individual also had a contiguous gene deletion, yielding a genetic diagnostic rate of 33% (11/33) across this second cohort [5]. In these studies, there was no evidence of recurrent point mutations and no genes which appeared to carry a higher burden of mutations, except for *SETBP1;* for which two individuals were found to carry variants across the two cohorts [6].

Taken together, the two cohort studies provided novel insights into the neurobiology of childhood speech disorders. First, the discovery of 17 new genes involved in CAS aetiology, with a combined diagnostic yield of 37%, revealing for the first time, that many children do have a single gene diagnosis explaining their speech condition. Second, many of the highly penetrant variants implicated shared pathways broadly involved in transcriptional regulation (e.g. *POGZ, SETBP1, SETD1A, KAT6A*), suggesting a key role for transcriptional dysregulation in aberrant speech development [4, 5]. Other molecular pathways of significance were also revealed with high confidence and likely novel pathogenic variants, such as in *GNAO1* and *GNB1*, both part of G-protein signalling pathways [5]. Third, the studies demonstrated that pathogenic variants more commonly arise de novo rather than being inherited, and that speech disorders are genetically heterogeneous [5], as is the case for other neurodevelopmental disorders [7–9]. Fourth, the candidate genes newly implicated in CAS were frequently associated with other neurodevelopmental disorders, such as epilepsy (e.g. *GNAO1, GNB1, SETD1A*) and/or intellectual disability (e.g. *CDK13*, *CHD3*, *DDX3X, POGZ, SETBP1*) [4, 5]. These novel insights into CAS aetiology, including genetic heterogeneity, demonstrate the need to study additional, larger cohorts to reveal further causative genes, increase the genetic diagnostic yield, and further unravel molecular pathways underlying severe childhood speech disorder. A much deeper knowledge of the molecular basis of severe speech conditions such as CAS is essential to move the field toward precision therapies.

Here, we aimed to identify the molecular basis of severe speech disorder, in a large cohort of probands ascertained for a primary diagnosis of CAS. Each proband underwent comprehensive phenotypic analysis and genome sequencing to identify pathogenic variants of major effect. We also analysed the molecular co-expression of all genes associated with CAS, and the overlap of genes associated with CAS and other neurodevelopmental disorders.

## Methods

### Ethical consent

The study was approved by the Human Research Ethics Committee of The Royal Children’s Hospital, Melbourne, Australia (#37353). Written informed consent was obtained from parents or legal guardians.

### Participants and phenotyping

Probands under age 18 years were ascertained with a clinical diagnosis of CAS and where parents and clinicians reported the current primary clinical concern as poor speech development due to childhood apraxia of speech [5]. Probands with moderate to severe intellectual disability as determined via psychometric testing, were excluded. Participants were recruited via medical and speech pathology clinicians or direct parent referral. Medical and developmental history and secondary neurodevelopmental outcomes were recorded with validation via relevant professional reports (e.g. paediatrician, multi-disciplinary assessment team for ASD diagnosis, physiotherapist, occupational therapist, academic outcomes) (Tables 1, 2; Supplementary Table 1).

**Table 1:** Medical and neurodevelopmental features of individuals with CAS and pathogenic/likely pathogenic variants.

Family	Age, y;m	Sex	Core speech phenotype	Gene	Gross motor delay	Fine motor delay	Vision impaired	Hearing loss	MRI findings	Seizures	Other NDD	Dysmorphic features		Other medical
1	16;8	F	CAS	*ARHGEF9*	Y	Y	Y	N	Small pineal cyst	Y	Mild ID, ASD	Clinodactyly 5th fingers*, severe class 3 malocclusion *with lateralisation of the mandible to the left	Prognathism and clinodactyly reported features	Cow milk allergy; selective IgA deficiency; polycystic ovaries; fibrous dysplasia of skull and jaw
2	5;6	F	Dysarthria	*DDX3X*	Y	Y	N	N	N	N	Mild ID, ASD, DCD	Upturned nose*, thin upper lip*, broad nasal tip*, small midface epicanthic folds		Gut inflammation, bowel issues, sleep disturbances
3	6;0	F	CAS	*KDM5C*	N	N	N	N	Hypoplastic cerebellar vermis and brainstem, possibly decreased volume of white matter, dysmorphic corpus callosum and hypoplastic hippocampi	N	Mild ID	Downslanting palpebral fissures*, low columella*, hypoplastic alae nasi		Severe allergic rhinitis with development of Harrisons sulci, tonsillectomy, adenoidectomy, obstructive sleep apnoea, grommets
4	7;4	M	CAS	*PHF21A*	Y	Y	N	N	N	N	Mild ID, attention difficulties, executive functioning difficulties	Hypoplasia of nasal septum, downturned corners of mouth*, supraorbital fullness*, smiling absent*		Eczema, adenoidectomy
5	3;2	M	Inconsistent phonological delay and disorder	*BRPF1*	Y	N	N	N	N	N	N	Periorbital fullness*, retrognathia, small cupped ears, thin lips, fine eyebrows, flat philtrum, downslanted palpebral fissures*		N
6	7;2	F	Phonological disorder, articulation disorder	*PURA*	Y	Y	Glasses	N	N	N	Attention difficulties	Pointed chin,* long face*		Oropharyngeal dysmotility
7	4;8	M	CAS	*ZBTB18*	Y	Y	Glasses	N	N	N	Attention difficulties	Wide mouth, everted lower lip, small jaw		Viral induced asthma; eczema; moderate oral phase dysphagia
8	3;2	F	CAS	*HNRNPK*	Y	Y	N	N	N	N	GDD	Slightly prominent forehead*, epicanthic folds, slightly broad nose*		Atrioventricular septal defect, hypotonia, grommets
9	5;9	M	CAS, phonological disorder	*SETD1A*	Y	Y	N	N	N	N	Sensory difficulties	High forehead, high anterior hairline		Undescended testis, sleep disturbances
10	10;1	M	CAS, phonological error patterns	*SETD1B*	Y	Y	Y	N	N	Y	N	Short philtrum*, anteverted ears*, prominent triangular nose*, small jaw		Low muscle tone, loose joints, short stature
11	4;3	M	CAS, phonological error patterns	*RBFOX3*	Y	Y	N	N	N	N	N	N		N
12	4;1	M	CAS, phonological error patterns	*TAOK2*	Y	Y	N	N	N	N	Mild ID, DCD	N		Collapsed lung
13	2;2	M	CAS, minimally verbal	*SPAST*	Y	N	N	N	N	N	N	N		Spastic diplegia
14	3;7	M	CAS, minimally verbal	*SHANK3*	Y	Y	N	N	N	N	N	N		N
15	7;4	M	CAS, phonological delay	*DIP2C*	Y	Y	N	N	N	N	N	N		N
16	3;11	M	CAS	*SETBP1*	Y	Y	N	N	N	N	Attention difficulties	Periorbital fullness*, broad nasal tip*, pointed chin		Motor dyspraxia, tongue tie (corrected)
17	4;6	F	CAS, Dysarthria	*TRIP12*	Y	Y	N	N	N/A	N	N	N		N
18	4;7	M	CAS	*ERF*	N	N	N	N	CHIARI 1 Malformation Metopic craniosynostosis	N	N	N		N

*Denotes dysmorphic features previously reported in the literature in association with the relevant gene.

*NDD* neurodevelopmental disorder, *ASD* Autism spectrum disorder, *CAS* childhood apraxia of speech, *DCD* developmental coordination disorder, *F* female, *ID* intellectual disability, *M* male, *N* feature not present, *SPD* sensory processing disorder, *Y* feature present.

**Table 2:** Linguistic phenotype and educational setting of individuals with CAS and pathogenic/likely pathogenic variants.

Family	Oral motor impairment	History of feeding issues	Language: receptive*	Language: expressive*	Reading deficits	Spelling deficits	Speech pathology	IQ	Education setting
1	Y	Y	Severe	Severe	Y	Y	Y	Mild ID (FSIQ 56)	Specialist
2	Y	Y	Severe	Severe	Y	Y	Y	Mild ID (FSIQ 69)	Mainstream (with classroom support)
3	Y	Y	Moderate	Severe	Y	Y	Y	Mild ID (FSIQ 68, VCI 80, VSI 73)	Mainstream
4	Y	N	Severe	Severe	Y	Y	Y	Mild ID (GMDS, 66)	Mainstream
5	Y	Y	Average	Moderate	TY	TY	Y	Borderline (KBIT IQ composite 72)	Mainstream
6	Y	Y	Severe	NA	Y	Y	Y	Borderline (PRI 78)	Mainstream (with classroom support)
7	Y	Y	Severe	Severe	TY	TY	Y	Borderline (FSIQ 70)	Mainstream
8	Y	Y	NS	NS	TY	TY	Y	Borderline (FSIQ 71)	Not yet at school
9	Y	N	Moderate	Moderate	Y	Y	Y	Borderline (FSIQ 79)	Mainstream
10	Y	N	Average	Average	Y	Y	Y	N (KBIT IQ Composite 109)	School for children with specific speech and language impairments
11	Y	N	Mild	Moderate	TY	TY	Y	N (WNV 95)	Mainstream
12	Y	N	Severe	Mild	TY	TY	Y	Mild ID (FSIQ 68, VCI 68, VSI 69)	Specialist
13	Y	N	Average	Severe	TY	TY	Y	NA^	Mainstream
14	Y	Y	Severe	Severe	TY	TY	Y	NA^	Mainstream
15	Y	N	Average	Moderate	Y	Y	Y	NA^	Mainstream
16	Y	N	Severe	Severe	TY	TY	Y	NA^	Mainstream
17	Y	Y	Moderate	Severe	TY	TY	Y	Borderline (FSIQ 71)	Mainstream
18	Y	N	Average	Average	TY	TY	Y	N (KBIT IQ Composite 89)	Mainstream

*FSIQ* full scale IQ, *N* no, *NA* not assessed, *VCI* verbal comprehension index, *VSI* visual spatial comprehension, *GMDS* Griffiths Mental Developmental Scales, *WNV* Weschler non-verbal scale of ability, *KBIT* Kaufman Brief Intelligence Test, *PRI* perceptual reasoning index, *TY* not applicable for literacy testing as pre-school age (<5 years old), *Y* feature present.

*Language severity rated according to CELF-5 [14] as follows: 86–114 average, 78–85 mild, 71–77 moderate, <70 severe.

^Assessment not previously indicated by the family or treating physician at time of study.

A diagnosis of CAS was then confirmed based on meeting the three American Speech-Language-Hearing Association consensus criteria for CAS: (1) inconsistent errors on consonants and vowels in repeated productions of syllables or words; (2) lengthened and disrupted coarticulatory transitions between sounds and syllables, and (3) inappropriate prosody [1]. Criteria were operationally defined and rated [10] from phonetic transcriptions of standardised single word speech sub-tests (phonology and inconsistency) [11] and a 5-min conversational speech sample [5]. Dysarthria was diagnosed in the presence of oral tone or coordination disturbance and dysarthric features identified during conversation using the Mayo Clinic Dysarthria rating scale [12, 13]. Language and cognition were also assessed with standardised tools [14–17].

### Genetic testing

Genomic DNA was extracted from whole blood or saliva using a Qiagen (Valencia, CA) QIAamp DNA Maxi kit or a prepIT L2P kit (DNA Genotek Inc., Ontario, Canada), respectively. Probands underwent chromosomal microarray testing on Illumina (SanDiego, CA) platforms, with the reportable effective resolution of arrays being 200 Kb. Results were analyzed with Karyostudio software version 1.3 or 1.4 (Illumina), using genome reference sequence NCBI36/hg18 (v1.3, pre-2013) or GRCh37/hg19 (v1.4, 2013 onwards).

Genome sequencing was conducted on 204 individuals from 70 families comprising 71 probands (two probands were monozygotic twins hence for the genetic analysis and results we report on 70 probands), 127 parents and 6 other relatives. Illumina TruSeq DNA Nano or NovaSeq PE150 PCR free library preparation was completed prior to sequencing on the Illumina NovaSeq 6000 to average 30-fold depth with ~100 Gb data generated per sample at the Australian Genome Research Facility or Novogene (HK) Company Limited. Sanger sequencing or droplet digital PCR (ddPCR) were used to segregate variants in additional family members who had not undergone microarray or genome sequencing.

### Variant analysis

**Fig. 1:**
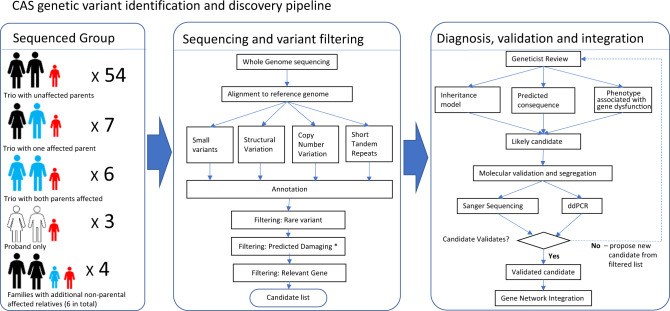
Genetic variant identification and variant filtering pipeline for individuals with CAS. Workflow covers recruitment of patients (CAS in red, affected relative in blue, unaffected in black, not sequenced in white), DNA sequencing, analysis and filtering of genomic data, identification of potential causative variants, geneticist review, molecular validation, segregation and integration of all findings. Please note that affectedness status refers to a parent having speech therapy as a child but not necessarily for a diagnosis of CAS which is not historically well reported for that generation. *Only the damaging effects of small intragenic variants are predicted bioinformatically.

Variant discovery was performed using trio or parent–child pair (where one parent was unavailable for testing) designs (Fig. 1). Exceptions to this were two singletons, and four larger families. 150 bp sequence pair-end reads were mapped to the hg19 reference genome using the Burrow-Wheeler Aligner (BWA-MEM, bwa v0.7.17) [18]. Read sorting and indexing was undertaken using SAMtools (v1.9) and Genome Analysis Toolkit (GATK, v4.1.4.1) was used to mark duplicates. Base quality score recalibration was performed, and variants were called using HaplotypeCaller, with individuals called separately, as implemented by GATK. Sequencing quality control was performed using fastQC.

Genotype calling and quality filtering were performed separately in multiple genome sequencing batches. Joint calling was performed by merging per-sample gvcf files and applying GATK’s GenotypeGVCFs tool. Variants with excess heterozygosity (Z score >4.5) were removed, then variant quality score recalibration was carried out for single nucleotide variants (SNVs) and indels separately, with a truth sensitivity filter of 99.7 to flag variants for exclusion. Filtering of low quality SNV calls excluded those flagged by low threshold or any of the following filters: low quality by depth (QD < 2); evidence of strand bias (Fisher strand [FS] > 60 or strand odds ratio [SOR] > 3); and evidence of significant differences between alternate and reference alleles for read mapping qualities (rank sum < −12.6) or position bias (ReadPosRankSum < −8). Indels filtering was performed in a similar manner to missense variant filtering, with exceptions being to exclude variants with FS > 200; SOR > 10; or ReadPosRankSum < −20. Finally, familial relationships were confirmed using peddy [19]. Filtering and other scripted analysis was conducted using R version 3.5.2.

Analysis was restricted to variants: (1) not present in gnomAD or with gnomAD allele count ≤2 (in all populations), (2) not present in unaffected family members from our cohort, and (3) potentially de novo, or consistent with an appropriate inheritance model matching the phenotypic pedigree (e.g. dominant, recessive). Compound heterozygous models were considered for variants present in gnomAD with a mean allele frequency <0.05%. Only variants with read depth >10 and genotype quality >20 in the proband and their sequenced family members were considered. Identified variants were annotated with the variant effect predictor (VEP v93.3) algorithm, using the assembly version GRCh37.p13 and categorized based on the following series of annotations.

### Genome-wide analysis of LoF and predicted damaging missense variants

We analyzed the genome sequencing data for loss of function (LoF) and predicted damaging missense variants genome-wide. Predicted LoF candidates were defined by using VEP annotations that were required to meet three criteria: (1) annotated as frameshift, stop or start lost, stop gained, splice acceptor or donor variant, (2) in a gene predicted intolerant to LoF variation (ExACpLI ≥ 0.9 or LoFtool <0.1), and (3) at least one of the following: (a) CADD Phred score ≥20 predicted damaging, or (b) predicted to affect splicing (AdaBoost score ≥0.6 or random forest score ≥0.6 using the dbscSNV VEP plugin). For frameshift variants, the variant was only required to be in a LoF intolerant gene.

Predicted damaging missense variants had to meet at least three criteria: (1) PolyPhen-2 prediction as “probably” or “possibly damaging”, (2) SIFT prediction as “deleterious” or “deleterious low confidence”, (3) a CADD Phred score ≥20 predicted damaging, or (4) a missense tolerance ratio significantly different from 1 (false discovery rate <0.05).

### Criteria for identification and reporting of candidate variants

We applied a two-stage approach for shortlisting candidate variants, from our identified LoF and damaging missense variants: (1) we selected variants located within genes of interest, a gene list collated based on several relevant criteria, informed by previous CAS studies [4, 5], and described below. Pathogenicity of these variants was assessed using the American College of Medical Genetics (ACMG) guidelines [20], and via review by a clinical geneticist; (2) where no candidate variant was identified, we then applied a genome-wide, wholly agnostic to gene, search for candidate variants, to be followed up with ACMG and clinical geneticist review. The size of the cohort and the inability to perform statistical analyses to implicate novel genes, such as via burden analysis, necessitated the usage of these constraints.

We report candidate variants as follows:

1. High-confidence variants: LoF and predicted damaging missense variants, that were classified with the American College of Medical Genetics (ACMG) guidelines as pathogenic (class 5) or likely pathogenic (class 4) [20], and where the phenotype associated with the gene was consistent with that of the proband.
2. Low confidence variants: LoF and predicted damaging missing variants, that were either classified as of uncertain significance (3) according to ACMG guidelines, or classified likely pathogenic (class 4), but where the gene was not consistent with the proband’s phenotype, or otherwise lacked evidence for pathogenicity.

All reported variants were inspected with the Integrative Genome Viewer (v 2.7).

### Collated list of genes of interest

The list of genes of interest, used in shortlisting candidate variants (*n* = 2145 genes, Supplementary Table 2), was collated from the following sources: genes from recent CAS cohort studies [4, 5] as well as previously confirmed single genes implicated in CAS such as *FOXP2* or *GRIN2A* [3, 21] and previously confirmed single genes associated with speech disorder or delay (*n* = 81). Additionally, high-confidence genes known to harbour pathogenic variants in intellectual disability (*n* = 1399), epilepsy (*n* = 611), autism spectrum disorder (ASD, *n* = 131) and cleft palate (*n* = 156), recognised by Victorian Clinical Genetics Services, were extracted from PanelApp using an application programming interface (https://panelapp.agha.umccr.org/) [22]. There were 233 overlapping genes across these groups making 2145 genes in total. High-confidence ASD-related genes from the Simons Foundation Autism Research Initiative database were also included [23] (*n* = 419). Finally, brain-expressed genes associated with primate-human accelerated evolution were included; this set comprised of 415 genes overlapping human accelerated regions (HARs) that are also significantly over-expressed in brain, compared to other tissues [24], and 45 genes overlapping with HARs, that were found to be exclusively expressed in human brain cells, and not in other primates [25]. This final set of genes were included, as HARs have previously been implicated in ASD and cognitive development, and thus may be involved in the evolutionary development of speech.

### Copy number and structural variants

Manta (regions up to 5 Mb) [26] (v 1.6.0) and qDNAseq (bin size 10 kb with CNVs up to 5 Mb) [27] (v 1.18.0) were used to detect CNVs and other structural variants. Manta detects structural variants based on abnormal alignment of read pairs. qDNAseq detects structural variants based on read depth. Variants occurring in more than two families were filtered out to avoid false positives due to technical artefact. SVAnnot (v 2.5) was used to annotate the variants, filtering by gnomAD SV abundance with SVs with frequency >0.05% excluded. Candidate structural variants were identified using the same approach as for SNVs, with pathogenicity assessed via ACMG guidelines and clinical review.

### Variant validation

High-confidence variants were validated using Sanger sequencing or ddPCR. For Sanger sequencing, gene variants were amplified using gene specific primers (oligonucleotide sequences available on request) designed to the reference human gene transcripts (NCBI Gene). Amplification reactions were cycled using a standard protocol on a Veriti Thermal Cycler (Applied Biosystems, Carlsbad, CA) at 60 °C annealing temperature for 1 min. Bidirectional sequencing of all exons and flanking regions was completed with a BigDye v3.1 Terminator Cycle Sequencing Kit (Applied Biosystems). Sequencing products were resolved using a 3730xl DNA Analyzer (Applied Biosystems). All sequencing chromatograms were compared to the published cDNA sequence; nucleotide changes were detected using Codon Code Aligner (CodonCode Corporation, Dedham, MA). For ddPCR, probes and primers were designed in-house and synthesized by Integrated DNA Technologies (Coraville, IA) and assays were performed [28, 29] using a Bio-Rad QX200 Droplet Digital PCR System (Hercules, CA) and QuantaSoft software v1.7.4.0917.

### Analysis of novel sources of genetic contributions to CAS

Three forms of genetic analysis for CAS that have not been previously applied were undertaken: (1) short tandem repeat (STR) analysis of both known and novel pathogenic repeats (Supplementary Table 3); (2) examination of common variants implicated in ASD and non-syndromic cleft palate, and their relevance to CAS, via associations with polygenic risk scores (PRS) and (3) estimation of mitochondrial gene abundance (see Supplementary methods).

### Brain gene co-expression and gene set enrichment analysis

Gene co-expression analyses were undertaken for our novel high-confidence genes identified in the present manuscript (*n* = 15) and then extended to include a further 19 genes previously implicated in cohorts ascertained for CAS across the Eising et al. [4]. (*CHD3*, *SETD1A*, *WDR5*, *KAT6A*, *SETBP1*, *ZFHX4*, *TNRC6B*, *MKL2, ARID1A, TRIO)* and Hildebrand et al. [5]. cohorts (*CDK13, EBF3, GNAO1, GNB1, DDX3X, MEIS2, POGZ, UPF2* and *ZNF142*), totalling 34 genes. *ARID1A* and *TRIO* are not yet confirmed candidate genes for CAS as parental data were not available, so de novo status and hence pathogenicity could not be confirmed in the original study [4, 20]. Yet both genes had previously been implicated in neurodevelopmental conditions where speech was a core phenotype and hence were included in expression analyses in Eising et al. [4] and also included in our meta-analyis here. Analyses were conducted using a Monte Carlo sampling approach [4, 5] with data from the BrainSpan Atlas of the Developing Human Brain [30] (Supplementary Table 4). Co-expression analyses were also performed to prioritize genes of uncertain significance for CAS (see Supplementary methods).

Gene set enrichment analyses were undertaken using gene sets in Gene Ontology molecular function, cellular component, and biological processes databases as well as Reactome pathway databases [31]. g:Profiler was used to test for gene set enrichment [32] with a Bonferroni-corrected *p*-value threshold of 0.05 to determine pathways enriched for genes implicated in CAS.

## Results

### Phenotypic data

**Fig. 2:**
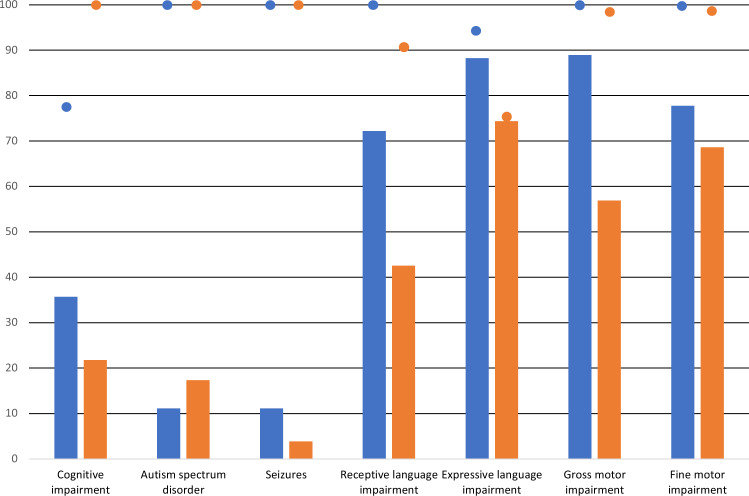
Phenotypic overlap in childhood apraxia of speech (CAS) cohort. Phenotypic features of CAS cohort with (blue, *n* = 18) and without (orange, *n* = 52) pathogenic variants. Data based on children with psychometric assessments by health professionals (i.e., cognition, language, motor, formal ASD diagnoses). Data from Tables 1, 2; Supplementary Tables 1a, b. Dots indicate percent of children with (blue) and without (orange) pathogenic variants who had psychometric test results confirming diagnoses.

One hundred and seventeen probands were recruited and 46 participants were excluded based on having an inappropriate phenotype (i.e. not having CAS; having another neurodevelopmental condition that affected the child’s development more than the speech presentation). The 71 included probands (53 males, 18 females, 1 monozygotic twin pair) from the 70 families had an average age of 5 years 7 months (range 2 years 2 months to 16 years 8 months). Phenotypic information for the cohort is shown in Tables 1, 2; Supplementary Tables 1a, b. Pathogenic variants were confirmed in 18 probands (12 males, 6 females; average age of 5 years 7 months) (Tables 1, 2) as described further in the following section. The phenotypes of the probands with (*n* = 18) versus without (*n* = 52) variants are presented in Fig. 2.

All probands were ascertained based on a clinical diagnosis of CAS. Following our speech assessment protocol, 15 of the 18 probands with pathogenic variants had CAS in isolation (*n* = 7) or co-occurring with other speech disorders (*n* = 8) (Table 1). Three probands (IDs 2, 5, and 6; Table 1) had other severe speech disorder presentations (dysarthria, *n* = 1; phonological and articulation disorder, *n* = 1; inconsistent phonological disorder, *n* = 1). Expressive language disorder was implicated in 15/17 probands (mild, *n* = 1; moderate, *n* = 4; severe or unable to be scored due to severity, *n* = 10; Fig. 2). One proband was minimally verbal and unable to complete a valid expressive language assessment. Receptive language disorder was noted in 13/18 individuals (mild, *n* = 1; moderate, *n* = 3; severe, *n* = 9). Of those old enough to read and write (>5 years, *n* = 8), all had reading and spelling impairment. Two probands with pathogenic variants (2/18, 11%; IDs 10, 18) had CAS accompanied by fine motor and related linguistic deficits, but without other neurodevelopmental disorder diagnoses.

Of the 18 children identified to carry high confidence variants, 14 had formal cognitive assessment with profiles ranging from an average full-scale IQ (FSIQ) (*n* = 3), to borderline FSIQ results (*n* = 6), to mild intellectual disability (*n* = 5). For 3 children, FSIQ could not be calculated due to significant performance variation across verbal and nonverbal subscales, which is a common experience for children with severe speech production deficits. The remainder (*n* = 4) did not have IQ testing because concerns with learning or cognition had not been raised or pursued by the family or treating physician and children were attending mainstream childcare or school settings (IDs 13–16; IDs 13, 14, 16 were <4 years of age when few children receive formal cognitive testing). Other features included neurodevelopmental diagnoses or features secondary to CAS including mild ASD (*n* = 2), difficulties with attention (*n* = 4), and anxiety and mood-related symptoms (*n* = 1). Dysmorphic features such as epicanthic folds and pointed chin (Table 1), rated by a clinical geneticist with 24 years of clinical experience, were present in just over half of the probands with high confidence variants (11/18). Gross motor (*n* = 16) and fine motor (*n* = 14) delays were common and associated with a slower trajectory in learning to ride a bicycle, balance appropriately, draw, write, and cut compared to typical peers. Two of the 18 children with pathogenic variants (IDs 1, 12) had a history of seizures.

### Single nucleotide and indel variants

**Fig. 3:**
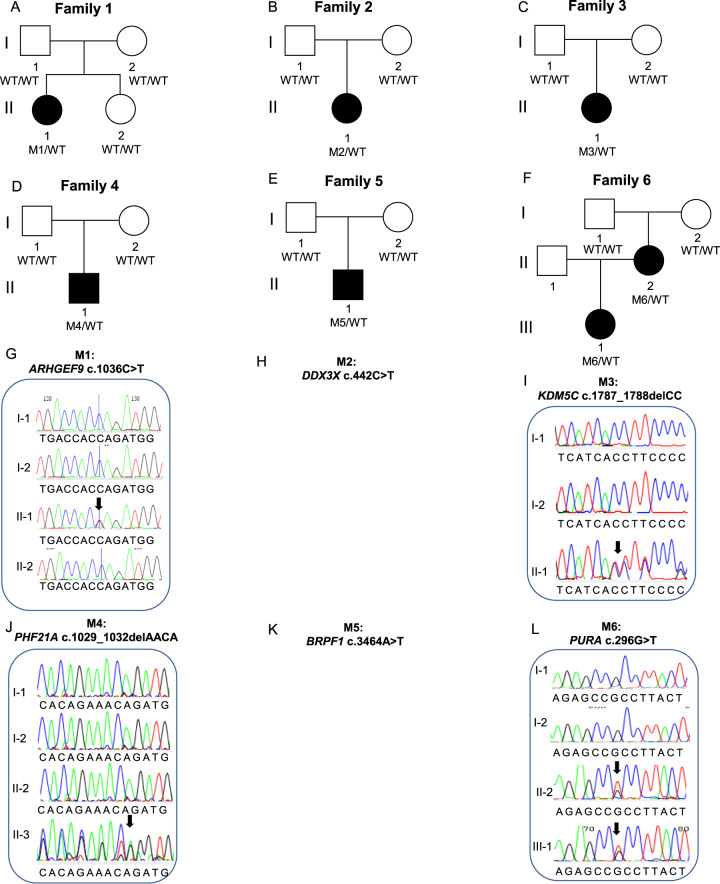

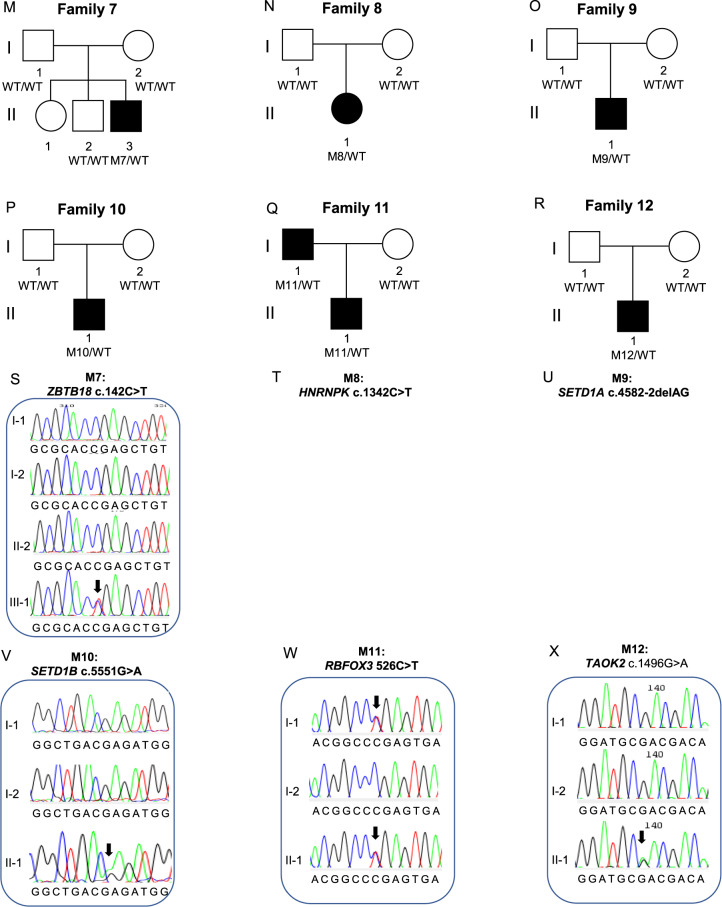

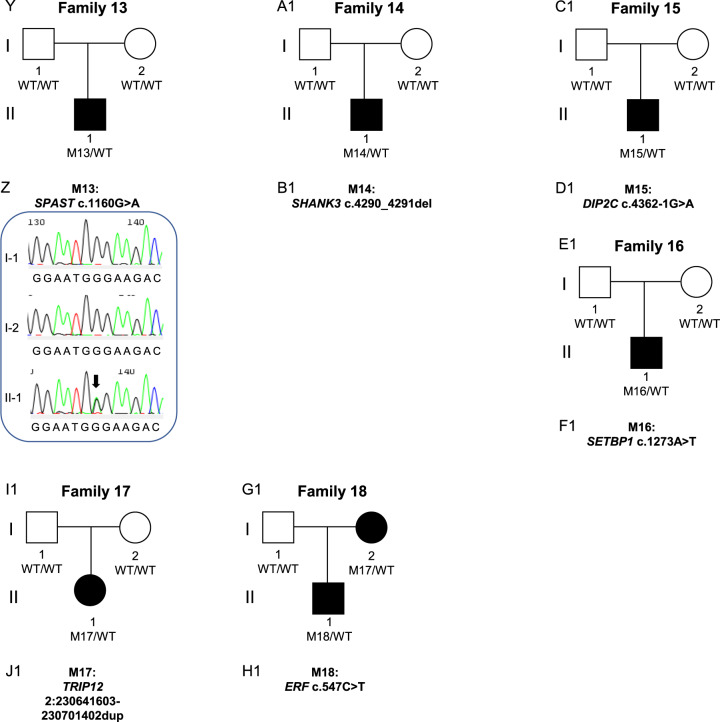
Families with high confidence variants analysed by genome sequencing. Families analysed by Genome Sequencing. Pedigrees (**A**–**F**, **M**–**R**, **Y**-**D**1) from 18 families with 18 different high confidence variants. Sequence chromatograms (**G**, **I**, **J**, **L**, **S**, **V**, **W**, **X**, **E**1) showing de novo or inherited variants. Sanger sequencing was not performed for the variants in eight of the families (**H**, **K**, **T**, **U**, **F**1, **G**1, **H**1, **I**1) because they had variants in known genes with sufficient coverage in the genome sequencing to be confident they were real, heterozygous variants. The large duplication in Family 17 (J1) could not be validated by Sanger sequencing.

**Fig. 4:**
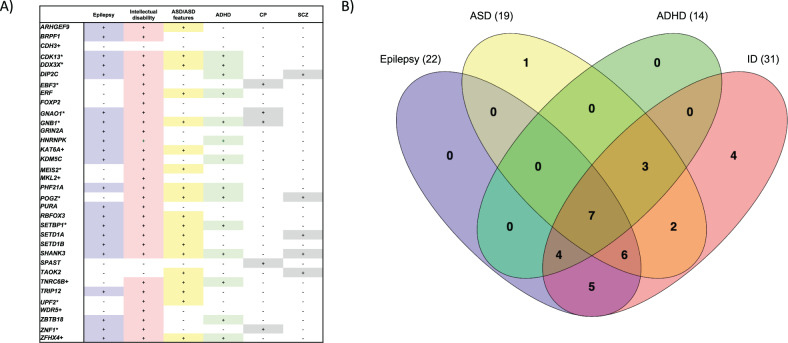
Previously identified neurodevelopmental conditions in candidate genes for CAS. **A** Candidate Genes for CAS identified in this study and Hildebrand et. al. (*) also have been shown to cause other neurodevelopmental disorder traits. **B** Venn diagram showing the overlap of these genes and multiple neurodevelopmental disorder traits.

A high confidence variant was identified in 18/70 (26%) of probands (Table 3a, Fig. 3). These included three frameshift, two splice acceptor, six nonsense, and six missense variants, as well as one multiple exon duplication, and they were found in 18 different genes (*ARHGEF9*, *BRPF1, DDX3X, DIP2C, ERF, HRNPNK, KDM5C, PHF21A, PURA, RBFOX3, SETBP1, SETD1A, SETD1B, SHANK3, SPAST, TAOK2, TRIP12, ZBTB18)*. All high-confidence variants were *de novo* except in *PURA, ERF* and *RBFOX3*, which were inherited (Table 3a, Fig. 3). Many of these genes that we newly implicate in CAS, as well as genes previously described in earlier sequenced CAS cohorts [4, 5] are also associated with other neurodevelopmental disorders (Fig. 4a and Fig. 4b, Supplementary Table 5).

**Table 3:** High and low confidence gene variants in the CAS cohort.

a: High-confidence gene variants in individuals with CAS.												
Family	Sex	Chr:Pos (GRCh37)	Gene (transcript)	OMIM	DNA Variant	Protein Change	Effect	In Silico Predictions^$^	gnomADCount ^~^	Inheritance	ACMG score	Reference*
1	F	X:62885786	*ARHGEF9* (*ENST00000253401*)	616056	c.1036 C > T	p.Q346*	Nonsense	gnomADpLI = 0.98;LoFtool = 0.0652;CADD = 38	0	De novo	PVS1, PS2, PM2, PP3 Class 5 Pathogenic	
2	F	X:41201905	*DDX3X (ENST00000399959)*	300958	c.442 C > T	p.Q148*	Nonsense	gnomADpLI = 1 LoFtool = 0.0555;CADD = 43	0	De novo	PVS1, PM2, PP3 Class 5 Pathogenic	
3	F	X:53231113	*KDM5C (ENST00000375401)*	300534	c.1787_1788delCC	p.T596IfsTer25	Frameshift	gnomADpLI = 1;LoFtool = 0.0636;	0	De novo	PVS1, PS2, PM2, PP3 Class 5 Pathogenic	
4	M	11:45975138	*PHF21A (ENST00000418153)*	608325	c.1029_1032delAACA	p.T344RfsTer28	Frameshift	gnomADpLI = 1;LoFtool = 0.08;	0	De novo	PVS1, PS2, PM2, PP3 Class 5 Pathogenic	
5	M	3:9788123	*BRPF1 (ENST00000383829)*	617333	c.3464 A > T	p.D1155V	Missense	SIFT = Del(0);PolyPhen = Dam (0.99);CADD = 33	0	De novo	PS2, PM2, PP3, BP1 Class 4 Likely Pathogenic	
6	F	5:139494062	*PURA (ENST00000331327)*	616158	c.296 G > T	p.R99L	Missense	SIFT = Del(0);PolyPhen = Dam (0.984);CADD = 28.7	0	Inherited from affected mother	PM2, PP1, PP2, PP3 Class 4 Likely Pathogenic	
7	M	1:244217218	*ZBTB18 (ENST00000358704)*	612337	c.142 C > T	p.R48*	Nonsense	gnomADpLI = 1 LoFtool = 0.18;CADD = 36	0	De novo	PVS1, PS2, PM2, PP3, PP5 Class 5 Pathogenic	[34]
8	F	9: 86585096	*HNRNPK (ENST00000376263)*	616580	c.1342 C > T	p.Q448*	Nonsense	gnomADpLI = 1 CADD = 43	0	De novo	PS2, PM2, PP3. Class 5 Pathogenic	
9	M	16:30992057	*SETD1A (ENST00000262519)*	619056	c.4582-2delAG	NA	Splice Acceptor Site	gnomADpLI = 1 LoFtool = 0.00654;CADD = 24.6	2	De novo	PVS1, PS2, PM2, PP3 Class 5 Pathogenic	[33]
10	M	12:122265722	*SETD1B (ENST00000604567)*	619000	c.5551 G > A	p.E1851K	Missense	SIFT = Del(0);PolyPhen = Dam (1);CADD = 33	0	De novo	PS2, PM1, PM2, PP2, PP3 Class 5 Pathogenic	
11	M	17:77097708	*RBFOX3 (ENST00000415831****)***	616999	c.526 C > T	p.R176*	Nonsense	gnomADpLI = 1 LoFtool = 0.17;CADD = 46	0	Inherited from affected father	PVS1, PM2, PP1 Class 5 Pathogenic	
12	M	16:29996607	*TAOK2 (ENST00000308893)*	613199	c.1496 G > A	p.R499Q	Missense	SIFT = Del(0.01);PolyPhen = Dam (0.992);CADD = 32	0	De novo	PS2, PM2, PP3, BP1 Class 4 Likely Pathogenic	
13	M	2:32352078	*SPAST (ENST00000315285)*	182601	c.1160 G > A	p.G387E	Missense	SIFT = Del(0); PolyPhen = Dam (1); CADD = 32	0	De novo	PS2, PM1, PM2, PP2, PP3 Class 5 Pathogenic	
14	M	22:51160325	*SHANK3 (ENST00000262795)*	606232	c.4290_4291del	p.V1432Gfs*4	Frameshift	gnomADpLI =1 CADD = 35	0	De novo	PVS1, PS2, PM2, PP3, PP5 Class 5 Pathogenic	[65]
15	M	10:323518	*DIP2C (ENST00000280886)*	611380	c.4362-1 G > A	NA	Splice Acceptor Site	gnomADpLI =1 LoFtool =0.356;CADD = 26.1	0	De novo	PVS1, PS2, PM2, PP3 Class 5 Pathogenic	
16	M	18:42530578	*SETBP1 (ENST00000282030)*	611060	c.1273 A > T	p.K425*	Nonsense	gnomADpLI = 1 LoFtool = 0. 0.0297;CADD = 38	0	De novo	PVS1, PS2, PM2, PP3 Class 5 Pathogenic	
17	F	2:230641603–230701402dup	*TRIP12 (NM_001348329)*	617752	NA	NA	Exonic duplication	gnomADpLI = 1	0	De novo	PVS1, PS2, PM2, PP3, PP5 Class 5 Pathogenic	
18	M	19:42753717	*ERF (ENST00000222329)*	617180	c.547 C > T	p.R183*	Nonsense	gnomADpLI = 0.99 LoFtool = 0. 0.02;CADD = 35	0	Inherited from affected Mother	PVS1, PM2, PP1, PP5 Class 5 Pathogenic	[36]

b: Low confidence, predicted LoF variants in individuals with CAS										
Family	Sex (M/F)	Chr:Pos (GRCh37)	Gene (transcript)	DNA Variant	Protein Change	Effect	In Silico Predictions^$^	gnomAD Count ^~^	Inheritance	ACMG score
34	F	X:71424983	*ERCC6L (ENST00000334463)*	c.3632_3633del	p.K1211Rfs*14	Frameshift	gnomADpLI = 0.99 LoFtool = 0.0101;CADD = 35	1	Maternal	PM2, PP3. Uncertain significance
39	M	15:44789223	*CTDSPL2 (ENST00000260327)*	c.771-2 A > G	NA	Splice Acceptor Site	gnomADpLI = 1 LoFtool = 0.0922;CADD = 34; adaScore = 0.999; rf_score = 0.924	0	Unknown	PM2, PP3. Uncertain significance
49	M	13:52532500	*ATP7B (ENST00000242839)*	c.2437_2458del	p.V761Pfs*39	Frameshift	LoFtool = 0.034	0	Inherited from Father	PM2, PP3. Uncertain significance
50	M	16:48247438	*ABCC11 (*ENST00000394747)	c.1271del	p.L424*	Frameshift	LoFtool = 0.015	0	Inherited from Father	PM2, PP3. Uncertain significance
56	M	20:62598776	*ZNF512B (ENST00000450537)*	c.221del	p.L74Rfs*18	Frameshift	gnomADpLI =1	0	Inherited from Father	PM2, PP3. Uncertain significance

c: Low confidence, predicted damaging Missense variants in individuals with CAS										
Family	Sex (M/F)	Chr:Pos (GRCh37)	Gene (transcript)	DNA Variant	Protein Change	Effect	In Silico Predictions^$^	gnomAD Count ^~^	Inheritance	ACMG score
19	M	4:160251101	*RAPGEF2 (ENST00000264431)*	c.758 C > T	p.M253T	Missense	SIFT = Del(0.01);PolyPhen = Ben (0.186);CADD = 23.2	0	*De novo*	PS2, PM2, PP3 Class 4 Likely Pathogenic
22	M	2:145156800	*ZEB2 (ENST00000558170)*	c.1954T > C	p.Y652H	Missense	SIFT = del(0.05), PolyPhen = PosDam(0.737), CADD = 25.9	0	Inherited from Father	PM2,PP3,BP1 Uncertain significance
28	M	8:38271461	*FGFR1 (ENST00000425967)*	c.2360 G > T	p.R787L	Missense	SIFT = del(0), PolyPhen = ProbDam(0.998), CADD = 33	0	Inherited from Father	PM1, PM2, PP2, PP3 Likely Pathogenic
30	M	X:56591124	*UBQLN2 (ENST00000338222)*	c.818 C > T	p.T273I	Missense	SIFT = del(0.01), PolyPhen = ProbDam(0.988), CADD = 26.9	0	Inherited from Mother - X-linked	PM2,PP2,PP3 Uncertain significance
43	M	8:77617377	*ZFHX4 (ENST00000521891)*	c.1054 A > G	p.N352D	Missense	SIFT = delLowConf(0.03), PolyPhen = ProbDam(0.991), CADD = 26	2	Unknown (parents not available)	PM2,BP1 Uncertain significance
50	M	5:88056897	*MEF2C (ENST00000340208)*	c.364 G > T	p.D122Y	Missense	SIFT = del(0), PolyPhen = ProbDam(0.998), CADD = 29.2	1	Inherited from Mother	PM2,PP2,PP3 Uncertain significance
50	M	2:74314988	*TET3 (ENST00000409262)*	c.2711 T > C	p.L904P	Missense	SIFT = del(0.01), PolyPhen = ProbDam(0.996), CADD = 25.9	0	Inherited from Father	PM1,PM2, PP2,PP3 Likely Pathogenic
52	M	3:77530328	*ROBO2 (ENST00000487694)*	c.625 G > A	p.V209M	Missense	SIFT = del(0.03), PolyPhen = ProbDam(1), CADD = 25.9	0	Inherited from Father	PM2,PP3,BP1 Uncertain significance
53	M	6:15497248	*JARID2 (ENST00000341776)*	c.1792G > A	p.E598K	Missense	SIFT = del(0), PolyPhen = Ben(0.225), CADD = 25.9	0	Inherited from Mother	PM2,PP3,BP1 Uncertain significance

*OMIM* Online Mendelian inheritance in man, *ACMG* American College of Medical Genetics, *NA* not applicable. All coordinates correspond to the Homo sapiens (human) genome assembly GRCh37 (hg19) from Genome Reference Consortium. ^$^In silico pathogenicity predictions: SIFT (sorting intolerant from tolerant), scores <0.05 reported, Del = ”Deleterious”; PolyPhen-2, scores >0.15 reported, Dam = ”Damaging”, PosDam = ”Possibly Damaging”; CADD (Combined Annotation Dependent Depletion), gnomADpLI (The Genome Aggregation Database (gnomAD) probability of intolerance to LoF), scores >0.9 reported; LoFTool, scores <0.1 reported. ^~^ Allele count (all populations) for variant from gnomAD or gnomAD SV for structural variants * Previously reported variant.

The 13 nonsense, splice-site, frameshift or exon duplication variants were all in genes intolerant to loss-of-function variation (*ARHGEF9*, *DDX3X, DIP2C, ERF, HNRNPK, KDM5C, PHF21A, RBFOX3, SETD1A, SETBP1, SHANK3, TRIP12, ZBTB18)*, according to gnomADpLI or LoFtool scores (Table 3a). The five missense variants were all predicted to be damaging by at least three in silico tools (SIFT, PolyPhen, CADD, MTR). Four of the 18 high confidence variants (in *ERF, SETD1A, SHANK3* and *ZBTB18)* were recurrent, with these variants having been reported previously in individuals with neurodevelopmental disorders [33–37]. The remaining 14 high-confidence variants have not been previously reported: of these, ten were pathogenic and four were likely pathogenic [20].

In five probands, we identified low confidence, LoF variants in genes predicted to be intolerant to haploinsufficiency (Table 3b, *ABCC11, ATP7B, CTDSPL2, ERCC6L, ZNF512B*). These variants are all predicted to cause loss of function of the gene; however at present, none of these genes are established to cause CAS or other neurodevelopmental disorders and therefore are variants of unknown significance. Nor did any of the cases have dysmorphology associated with any of these conditions. Of note, a frameshift variant in *ATP7B* of uncertain significance was also identified in our previous CAS cohort [5]; in the present cohort, the identified variant (proband 49) is shared with their father; however the father has a history of self-reported but undiagnosed dyslexia without CAS, and the mother has a brief history of speech therapy as a child but also without a CAS diagnosis, so the variant does not fully segregate with CAS or speech affected status. Thus, the relevance of *ATP7B* in CAS remains unclear.

In eight probands (8/70; 11.4%), we report nine rare (gnomAD allele count < =2) low confidence, predicted damaging missense variants (Table 3c). These are a selected subset of predicted damaging variants, located in genes that were of relevance due to known disease association, or biological relevance, but were of uncertain pathogenicity, or the gene was not consistent with the proband’s phenotype. Two of the nine variants were in genes previously associated with speech and/or language disorders - *ROBO2*, and *ZFHX4*; however, one of these was inherited from an unaffected parent (*ROBO2*, Proband 52), and for the other, it was not possible to determine whether the variant was de novo, as parental DNA was unavailable (*ZFHX4*, proband 43). The remaining seven variants were located in *CHD5*, *FGFR1*, *JARID2*, *MEF2C*, *RAPGEF2, TET3*, *UBQLN2* and *ZEB2*. For each of these, the variant was deemed to be of uncertain significance (ACMG), and/or the phenotype associated with the gene was not consistent with the proband’s phenotype. For all probands without a high confidence variant, the remaining predicted damaging missense variants, identified though our genome-wide search, are listed in Supplementary Table 6.

### Copy number and structural variation

There were no diagnostic findings on clinical microarray analysis. In one individual (ID17) a *de novo* pathogenic tandem 59,799 bp duplication was identified in *TRIP12*, spanning exons 7 to 37 out of 42 (NM_001348323.3). If tandem, this duplication would be predicted to disrupt the reading frame causing loss of function (Supplementary Fig. 1), however we could not confirm this using the microarray probe data and independent confirmation by sequencing would be required.

### Analysis of novel sources of genetic contributions to CAS

No expansion of either known or novel repeats were identified in the CAS probands. The polygenic risk score analysis did not identify any statistically significant findings, with the strongest trend being observed for ASD where probands were enriched for ASD risk, nearly achieving nominal significance (Two sample *t* test, *p* = 0.054). The non-syndromic cleft palate PRS also showed increased risk for CAS probands, but this was not significant (two sample *t* test, *p* = 0.226).

Mitochondrial abundance analyses identified two CAS probands with high confidence, likely pathogenic variants in genes known to have mitochondrial function as outliers (*DDX3X* and *HNRNPK*) but mitochondrial abundance did not appear to be a biomarker for CAS overall (see Supplementary results, Supplementary Fig. 2A–C).

### Brain gene co-expression and gene set enrichment analyses

**Fig. 5:**
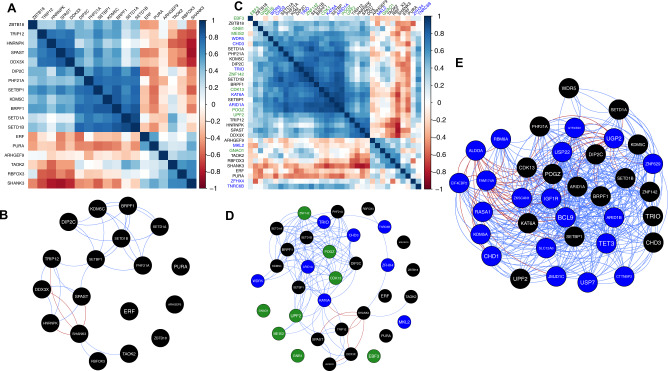
CAS candidate gene co-expression. **A** Gene co-expression matrix for the 18 high-confidence candidate genes with pairwise Spearman’s rank correlation coefficients between genes shown, based on 280 samples from 24 individuals (8 weeks post conception to 10 months after birth) from the BrainSpan resource. Genes ordered by hierarchical clustering, using the median linkage method. **B** Network of gene co-expression. Nodes represent genes; edges represent gene–pair correlations that exceed the threshold for the top 5% most highly correlated gene pairs genome-wide (|ρ | > 0.64) (blue—positive correlation, red—negative correlation). **C** Gene co-expression matrix for the 18 high-confidence candidate genes (black) as well as the genes from [4] (green) and [5] (blue). **D** Network of gene co-expression. Nodes represent genes; edges represent gene–pair correlations that exceed the threshold for the top 5% most highly correlated gene pairs genome-wide (|ρ | > 0.64) (blue—positive correlation, red – negative correlation). Black nodes—novel genes from this work, green nodes genes from [4] and blue nodes from [5]. **E** Network of gene co-expression. Nodes represent genes; edges represent gene–pair correlations that exceed the threshold for the top 5% most highly correlated gene pairs genome-wide (|ρ | > 0.64) (blue—positive correlation, red—negative correlation). Black nodes are a set of co-expressed genes including genes from the present study and from previous studies [4, 5]. Blue nodes—the top prioritized genes from cytogenic variants described in Table 4.

The median absolute correlation between our 18 high-confidence genes was |*ρ* | = 0.4194 (Fig. 5A). Thirty-two of the 153 pairwise correlations were among the top 5% most highly correlated gene pairs genome-wide |*ρ* | > 0.647, (Fig. 5B), and there was evidence that this set of genes was more highly co-expressed than expected by chance (*p* = 0.0038). Gene set enrichment analyses of a subset of seven highly co-expressed genes (*BRPF1, DIP2C, KDM5C, PHF21A, SETBP1, SETD1A, SETD1B*) indicate they are involved in chromatin organization (GO:0006325; *p* = 1.238 × 10^–3^). (Supplementary Table 7)

The median pairwise correlation of gene expression for the 34 genes, was significantly higher than expected by chance (median | ρ | = 0.4095, *p* = <2 × 10^–4^, Fig. 5D). Gene set enrichment analyses of the highly co-expressed cluster of 15 genes from the present study and past cohorts [4, 5] (Fig. 5C) further highlighted the significant over-representation of genes involved in chromatin organization (GO:0006325; Bonferroni-corrected *p* = 2.304 × 10^–6^) as well as transcriptional regulation (GO:0003676; Bonferroni-corrected *p* = 1.103 × 10^–4^, 25,396 sets tested, Supplementary Table 7).

Finally, our co-expression model was used to prioritize candidate genes, beyond our high confidence set. Firstly we re-examined the set of low-confidence variants identified in the present study (Table 3b, c), and variants of uncertain significance from our previous cohort [5]. Amongst the low confidence findings, *TET3* was the only gene identified for proritization (FDR < 0.1), while three genes identified in our previous cohort were prioritized (*BRWD3, MCMBP* and *ZKSCAN1*). All four prioritzed genes are associated with chromatin organization and/or DNA binding. Second, we sought to prioritize genes contained in each of 21 large copy number variant regions, identified through a literature search. All regions span multiple genes, and the associated phenotypes include speech disorder as a clinical feature (Supplementary Tables 8 and 9). Prioritization analysis identified at least one gene in each region (FDR < 0.1), with more than one candidate for 18/21 regions (Table 4) (Fig. 5E). In several instances, the prioritized gene from our co-expression network had already been proposed as the likely causal gene (see Supplementary results).

**Table 4:** Genes prioritized in cytogenetic regions which have previously been identified with speech and language disorders.

Region	Prioritized genes
1q21.1 microdeletion	*BCL9* (13.4)
2p15	*UGP2* (8.6), *USP34* (5.9), *PEX13* (5.3)
5q14.3	*RASA1* (8.1)
5q14q21.1	*CHD1* (7.4)
6q25.3	*ARID1B* (12.2), *TULP4* (6.9)
7q11.23	*GTF2IRD1* (11.5), *BAZ1B* (10.1), *RSBN1L* (8.4), *GTF2I* (7.6), *RHBDD2* (5.7)
7q31.1	*CTTNBP2* (5.5)
7q31.2-q31.31	*CTTNBP2* (5.5)
10q21.2–22.1	*JMJD1C* (8.5), *CISD1* (8.1)
15q14	*SLC12A6* (10.6), *ANP32AP1* (5.7)
12p13.33-p13.32	*KDM5A* (8.9), *ERC1* (8.1)
15q14	*SLC12A6* (10.6), *ANP32AP1* (5.7)
15q26.3	IGF1R (11.2)
16p11.2	*ZNF629* (11.5), *SRCAP* (10.8), *ZNF764* (10.5), ZNF646 (10.4), *SETD1A* (9), *ZNF48* (6.9), *ALDOA* (6.4), *ATXN2L* (5.8)
16p11.2 microdeletion	*ALDOA* (6.4)
16p13.2	*USP7* (6.9)
17p11.2	*USP22* (10.8), *MAPK7* (10), *TOP3A* (9.4), *SMCR8* (8.9), *RAI1* (8.3), *DHRS7B* (7.9), *TRPV2* (6.7)
19q13.11	*GPATCH1* (8.8), *CEP89* (8.3), *SLC7A10* (6.4), *ZNF507* (6.1)

Genes listed are those within each cytogenetic region including those known to be deleted whose prioritization modelling based on known candidate genes and neurodevelopmental gene co-expression are predicted to have a similar loss of function phenotype. Some genes are in multiple regions as they overlap in the genome. Number in parentheses represents the continuous connectivity score. See Supplementary Table 8 for full data set including references and data sources.

## Discussion

Our findings almost double the current number of genes implicated in causation of CAS and provide further novel insights into the biology of childhood speech disorder. We identified high confidence variants, thereby providing a clinical genetic diagnosis, for 18 children ascertained for CAS, revealing 15 genes that have not previously been associated with this severe speech disorder (*ARHGEF9*, *BRPF1, DDX3X, DIP2C, ERF, HRNPNK, KDM5C, PHF21A, PURA, RBFOX3, SETBP1, SETD1A, SETD1B, SHANK3, SPAST, TAOK2, TRIP12, ZBTB18*). We identified a clinical genetic diagnosis in one-quarter of individuals tested; a diagnostic yield comparable to or even higher than other neurodevelopmental disorders with a substantial burden of de novo variants [38]. We provide independent confirmation with unrelated cases for three genes previously implicated in CAS; *SETD1A* [4], *DDX3X* [5] and *SETBP1* [5, 6]. We highlight chromatin organization and transcriptional regulation as critical biological mechanisms underpinning speech development.

The high confidence variants in this study were all located in genes previously associated with other common neurodevelopmental phenotypes including epilepsy, intellectual disability and ASD [4, 5]. These complex speech and neurodevelopmental presentations match our current understanding of genes that have been associated with ASD, epilepsy and/or intellectual disability, where pleiotropy, or overlapping comorbid phenotypes, are common [39]. However, for 15 of the 18 genes, this is the first time they have been specifically associated with CAS. Our work highlights the current bias in the literature to gene discovery cohorts across intellectual disability, autism and epilepsies relative to speech disorder. Although there is arguably some circularity here because our variant curation pipeline did prioritise variants previously associated with neurodevelopmental disorders. Still, we have expanded the phenotypic spectrum for a number of genes previously implicated in neurodevelopmental disorders, linking them with specific speech diagnoses, as well as markedly increasing the list of genes that should be prioritized for clinical testing in individuals with CAS.

Probands for whom we could provide a genetic diagnosis had a higher proportion of motor, language and cognitive impairments, secondary to the primary concern of CAS, compared to those probands without genetic diagnoses at a group level. We provide preliminary evidence for a threshold effect where monogenic conditions may be more likely when individuals with CAS have additional neurodevelopmental conditions, although further work on larger cohorts is needed to confirm this hypothesis. Only two probands (11%) with genetic diagnoses (*SETD1B* (ID10)*, ERF* (ID18)) had CAS without co-occurring neurodevelopmental disorder diagnoses. One was aged 10;8 years, had average IQ and was attending a school for children with specific speech and language impairment. The other child was only 4;7 years and had not yet had IQ testing because no concerns had been raised by his treating physician, family or preschool teacher regarding his general learning ability; however, it is possible that other neurodevelopmental diagnoses could still be made into the future.

These findings expand the spectrum of phenotypes associated with these conditions. *SETD1B* has been previously associated with epilepsy, intellectual disability and language delay, and *ERF*-related craniosynostosis syndrome often includes speech and language delay, learning difficulties or behavioural problems; however variable expressivity and incomplete penetrance have previously been observed [40]. Our data suggest that monogenic causes can underpin the less commonly occurring more “specific” CAS phenotypes. We also reinforce the observation that, just as recent reports have suggested there are no ‘autism-(specific) genes,’ [39] it appears “speech-specific” monogenic conditions are also rare. This has recently been acknowledged for individuals with *FOXP2* variants, where the phenotypic spectrum has been expanded from a relatively specific speech condition to include learning difficulties in at least some of the affected individuals [41, 42]. These observations of neurodevelopmental phenotypic comorbidity across genetic conditions and in our own data imply that it may be short-sighted to exclude children with autism, epilepsy or moderate to severe intellectual disability from studies examining the genetic basis of CAS.

In terms of the biological pathways associated with speech disorder, we found a significant over-representation of perturbed chromatin and transcriptional regulation pathways, consistent with prior studies [4, 5]. The five chromatin-related genes habouring high confidence variants in the current cohort were significantly co-expressed during brain development and are co-expressed with similar genes previously implicated in CAS[4, 5]. *KDM5C* encodes a histone demethylase involved in regulation of gene expression [43] and DNA methylation [44], and LoF mutations in this gene have been shown to cause intellectual disability in females [45]. *BRPF1*, encoding a histone acetyl transferase, has also been associated with intellectual disability and dysmorphic features [46]. *SETD1A* encodes a histone methyltranferase and has previously been associated with schizophrenia, intellectual disability, and speech and/or language delays [33]. *SETD1B* is also a histone methyltransferase associated with neurodevelopmental disorder [47]. *PHF21A* is a member of the *BRAF35*/histone deacetylase complex that mediates repression of neuron-specific genes [48] and has previously been associated with ASD and intellectual disability [49]. *HNRNPK* encodes an RNA-binding protein known to interact with many molecular partners in multiple processes that regulate gene expression: chromatin remodelling, transcription, and mRNA splicing, translation, and stability [50]. De novo truncating variants in *HNRNPK* have been shown to cause Au-Kline neurodevelopmental syndrome including intellectual disability, ADHD, speech impairment, cardiac anomalies and a variety of dysmorphic features [37]. These results support earlier findings [4, 5] that chromatin modifiers and transcriptional regulators are critical for speech development. More generally, chromatin modifiers play important roles in neurodevelopmental disorders as they are key regulators of progenitor expansion, differentiation, cell-type specification, migration and maturation, with early errors in chromatin remodelling known to impact development of brain networks [51].

Other genes confirmed to harbour pathogenic variants in CAS and associated with chromatin organization and transcriptional regulation were not as highly co-expressed during brain development. Among these, *DDX3X*, regulates gene splicing and is associated with neurodevelopmental disorders characterised by intellectual disability, ASD [52] and more recently, CAS [5]. *RBFOX3*, a gene showing neuron-specific expression, is also involved in splicing and has been associated with epilepsy aphasia syndrome and impaired language [53]. *PURA* is implicated in the control of both DNA replication and transcription [54], and PURA syndrome is noted to include ‘absent speech’ as a feature [55]. *ZBTB18* encodes a transcriptional repressor shown to play a critical role in orchestrating brain development, and has been associated with non-syndromic intellectual disability [56].

The remaining candidate genes that we newly implicate in CAS (*TAOK2, SPAST* and *ARHGEF9*) encode proteins with distinct functions. *TAOK2* is located in the 16p11.2 deletion region, a well-recognised CNV associated with CAS, among other neurodevelopmental phenotypes [10, 57]. The protein encoded by *TAOK2* has established roles in dendrite growth and synapse development [58]. *ARHGEF9* encodes collybistin, a brain-specific guanine nucleotide exchange factor. The gene has also been implicated in X-linked epileptic encephalopathy and neurodevelopmental disorder, where there is skewed X-inactivation in favour of the abnormal X-chromosome [59]. Finally, *SPAST* promotes microtubule growth in the cytoskeleton, playing an important role in neuronal development, and has been implicated in spastic paraplegia with dysarthric speech [60]. Although previous reports describing individuals with *SPAST* variants have not thoroughly characterised speech in the early years of life, it is possible that CAS was part of the early profile of such cases. A pattern of CAS alongside dysarthria is not uncommon in other genetic forms of persistent speech disorder, for example in Koolen de Vries Syndrome [61], *SETBP1*-haploinsufficiency disorder [6], or *EBF3*-related core motor disturbance and ataxia [5].

Whilst we were able to attain a diagnostic rate of 26%, other highly penetrant risk variants may have remained hidden due to our strict definition of high confidence variants, which was largely based on ACMG guidelines. By definition, for an identified variant to be deemed high confidence, a causative link to a relevant disorder must be already established. Hence it is likely that a proportion of our low confidence candidate variants are truly causal but currently lack sufficient evidence. Identification of additional variants in these low confidence genes in future cohorts of individuals with CAS would elevate thes findings to declare these genes as truly implicated in CAS. Currently, they provide candidate genes for future studies. Our filtering strategy may also have been overly conservative for genes harbouring variants with a recessive mode of inheritance. For example, we utilised pLI scores for prioritising LoF variants; these scores are relevant identifying genes that are intolerant of heterozygous protein truncating variants, but may be less appropriate for identifying genes that are intolerant to homozygous variants. We also performed an expanded variant identification analysis including short tandem repeats which yielded no hits, suggesting at this time, that they do not play a major role in CAS [62, 63].

Our data confirm that a substantial proportion of children with CAS or equally marked and persistent speech disorders may have a monogenic condition. As such CAS can be viewed as a critical clinical indicator for single gene disorders, due to its sensitivity as a rare phenotype (1 per 1000) [4], relative to more common speech diagnoses such as articulation or phonological disorder (1 in 20) [64]. While some individuals may have relatively ‘specific’ CAS in the absence of other neurodevelopmental disorders, our findings support the increasing overlap between genes conferring risk for a range of neurodevelopmental disorders including CAS, epilepsy, ASD and intellectual disability. This observation is important because well defined speech diagnoses are not typically reported in published clinical studies where the focus lies on other diagnoses like intellectual disability, epilepsy or ASD. If there is mention of speech or language impairment, the phrase ‘speech delay’ is typically used, which is a highly non-specific term that could imply general language understanding or expression difficulties (e.g. in semantic or syntactic domains), and hence may not even be referring to ‘speech’ impairment itself (e.g. difficulty with producing speech sounds) [12]. Thus, our work highlights the importance of specifically describing speech and language phenotypes, that is, at the very least being specific with the presence of clinical speech diagnoses of phonological disorder, stuttering, CAS and dysarthria, and specifying whether language is also impaired, and if so, in what domains, and by conducting genotype-phenotype correlation studies. We also propose considering a core diagnosis of CAS as a red flag for a monogenic condition. Understanding the aetiological basis of CAS is critical to end the diagnostic odyssey, identify comorbidities and ensure patients are poised for precision medicine trials.

## Supplementary information

Supplemental methods and results

Supplemental Table 1

Supplementary Tables 2 to 9

## Data Availability

Data can be made available by contacting the corresponding author.
