# Correction: Genetic aetiologies for childhood speech disorder: novel pathways co-expressed during brain development

**DOI:** 10.1038/s41380-022-01879-y

**Published:** 2023-01-19

**Authors:** Antony Kaspi, Michael S. Hildebrand, Victoria E. Jackson, Ruth Braden, Olivia van Reyk, Tegan Howell, Simone Debono, Mariana Lauretta, Lottie Morison, Matthew J. Coleman, Richard Webster, David Coman, Himanshu Goel, Mathew Wallis, Gabriel Dabscheck, Lilian Downie, Emma K. Baker, Bronwyn Parry-Fielder, Kirrie Ballard, Eva Harrold, Shaun Ziegenfusz, Mark F. Bennett, Erandee Robertson, Longfei Wang, Amber Boys, Simon E. Fisher, David J. Amor, Ingrid E. Scheffer, Melanie Bahlo, Angela T. Morgan

**Affiliations:** 1 Population Health and Immunity Division, The Walter and Eliza Hall Institute of Medical Research, Parkville, VIC 3052 Australia; 2 Faculty of Medicine, Dentistry and Health Sciences, University of Melbourne, Parkville, VIC 3052 Australia; 3 Murdoch Children’s Research Institute, Parkville, VIC 3052 Australia; 4 Neurology Department, The Children’s Hospital at Westmead, Westmead, NSW 2145 Australia; 5 Queensland Children’s Hospital, South Brisbane, QLD 4101 Australia; 6 University of Queensland, St. Lucia, Brisbane, QLD 4067 Australia; 7 Hunter Genetics, John Hunter Hospital, New Lambton Heights, NSW 2305 Australia; 8 School of Medicine and Menzies Institute for Medical Research, University of Tasmania, Hobart, TAS Australia; 9 Tasmanian Clinical Genetics Service, Hobart, TAS Australia; 10 Royal Children’s Hospital, Flemington, Parkville, Melbourne, VIC Australia; 11 University of Sydney, Camperdown, NSW 2006 Australia; 12 Griffith University, Gold Coast, QLD Australia; 13 Victorian Clinical Genetics Services, Parkville, VIC Australia; 14 Language and Genetics Department, Max Planck Institute for Psycholinguistics, 6525 XD Nijmegen, The Netherlands; 15 Donders Institute for Brain, Cognition and Behaviour, Radboud University, 6500 HE Nijmegen, The Netherlands

**Keywords:** Genetics, Molecular biology

Correction to: *Molecular Psychiatry* 10.1038/s41380-022-01764-8, published online 18 September 2022

Wording was altered for the discussion.

Only two probands (11%) with genetic diagnoses (*SETD1B* (ID10), *ERF* (ID18)) had CAS without co-occurring neurodevelopmental disorder diagnoses. One was aged 10;8 years, had average IQ and was attending a school for children with specific speech and language impairment. The other child was only 4;7 years and had not yet had IQ testing because no concerns had been raised by his treating physician, family or preschool teacher regarding his general learning ability; however, it is possible that other neurodevelopmental diagnoses could still be made into the future. These findings expand the spectrum of phenotypes associated with these conditions. SETD1B has been previously associated with epilepsy, intellectual disability and language delay, and ERF-related craniosynostosis syndrome often includes speech and language delay, learning difficulties or behavioural problems; however variable expressivity and incomplete penetrance have previously been observed [40].

See attached files for table and figure changes.

**Table 1** 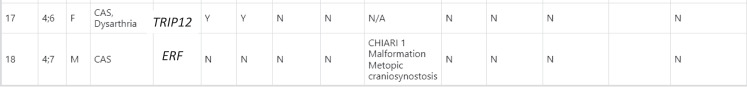


**Table 3a** 
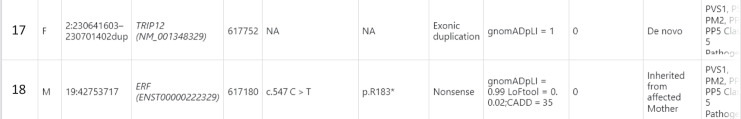


**Fig. 3** 
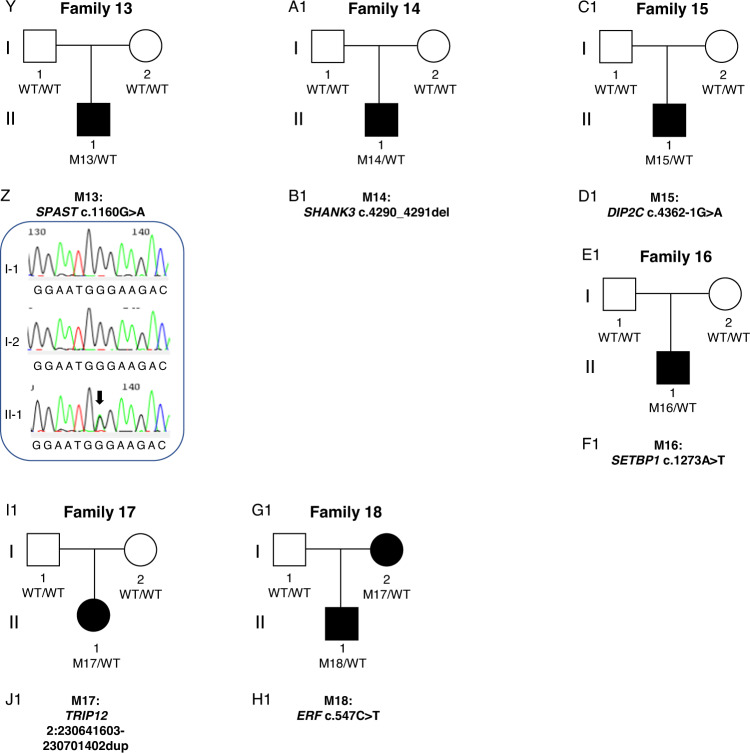


**Fig. 4** 
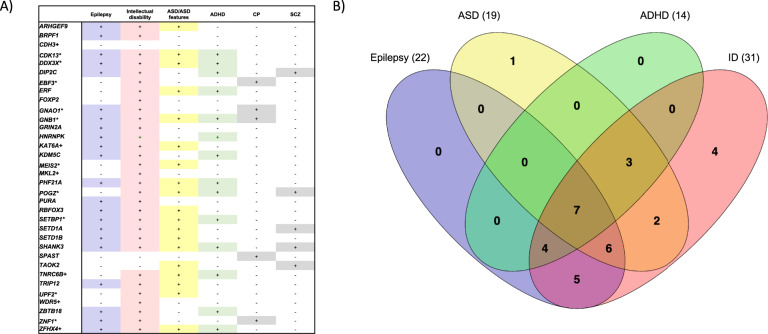


The original article has been corrected.

