# Supplementary material for: Genetic aetiologies for childhood speech disorder: novel pathways co-expressed during brain development
Source: Mol Psychiatry. 2022 Sep 18;28(4):1647–63. doi: 10.1038/s41380-022-01764-8 (PMC10208970; doi:10.1038/s41380-022-01764-8)
Supplement: Supplementary file 2 — Supplemental Table 1 [file 41380_2022_1764_MOESM2_ESM.docx]

Supplementary Table 1a - Medical and neurodevelopmental features of patients without confirmed variants

| **Family** | **Age, y;m** | **Sex** | **Core speech phenotype** | **Gross motor delay** | **Fine motor delay** | **Vision impaired** | **Hearing loss** | **MRI findings** | **Seizures** | **Other NDD** | **Dysmorphic features** | **Other medical** |
| --- | --- | --- | --- | --- | --- | --- | --- | --- | --- | --- | --- | --- |
| **19** | 5;2 | M | CAS, phonological delay | Y | Y | N | N | N | N | DCD, SPD | N | Benign joint hypermobility, anxiety |
| **20** | 5;8 | F | CAS | NA | NA | N | N | NA | N | N | Hypotelorism, deep set eyes, thin lips with downturned corners of mouth, low columella | NA |
| **21** | 4;4 | M | Phonological disorder | Y | Y | N | N | NA | N | GDD | Prominent forehead | N |
| **22** | 6;2 | M | CAS | N | N | N | N | NA | N | N | N | N |
| **23** | 4;10 | M | CAS | Y | Y | N | N* | NA | N | ASD | N | Generalised hyperextensibility, sleep disturbances |
| **24** | 6;4 | M | CAS | Y | Y | N | N | NA | N | ID | N | N |
| **25** | 13;10 | F | CAS | Y | Y | Y | N | NA | N | ID | N | N |
| **26** | 12;2 | M | CAS, Dysarthria | Y | Y | Y | N | N | N | N | Long face, tall forehead, slightly deep-set eyes, high palate | Congenital hypotonia, constipation |
| **27** | 6;10 | M | CAS | N | N | N | N | NA | N | N | Periorbital fullness,  ptosis | N |
| **28** | 4;7 | M | CAS, Phonological disorder | N | N | N | N* | NA | N | ASD | N | Hypermobile |
| **29** | 7;8 | F | CAS | Y | Y | N | N* | NA | N | ID | Prominent forehead, mild mid-face hypoplasia, short philtrum, thin nose | N |
| **30** | 5;0 | M | CAS | Y | Y | N | N | NA | N | N | N | N |
| **31** | 3;1 | F | Dysarthria, phonological disorder, history of stuttering | Y | Y | Y | Y (conductive hearing loss) | Bilateral optic nerve sheath distention | N | N | N | Stenosis left branch pulmonary artery, Immunoglobulin deficiency, possible common variable immune deficiency, left lower limb spasticity with catch in left ankle, catch right ankle mild, chronic constipation, neonatal hepatitis, right side hemiplegia |
| **32** | 5;4 | F | CAS | N | Y | N | N | NA | N | N | N | Twin |
| **33** | 6;9 | M | CAS | Y | Y | N | N | NA | N | ASD | N | Twin |
| **34** | 7;0 | F | CAS | Y | Y | N | N | NA | N | ASD | N | Anxiety, hashimotos thyroiditis |
| **35** | 10;9 | M | CAS | Y | N | Y | N* | NA | N | ASD, DCD | Hypoplastic alae nasi, slightly anteverted ears | Sleep disturbance, asthma, eczema, anxiety |
| **36** | 5;2 | M | CAS | N | N | N | N | Increased T2 signal in left thalamocapsular region | Epilepsy aphasia spectrum, benign childhood epilepsy with centrotemporal spikes. | ASD | N | N |
| **37** | 3;8 | M | CAS | Y | Y | N | N* | Normal | N | GDD | Flat in midface, pointed chin, slightly downslanted palpebral fissures, broad forehead, deep set eyes, camptodactyly of toes with thick toenails | N |
| **38** | 4;4 | M | CAS | N | N | N | N | NA | N | N | NA | N |
| **39** | 6;0 | M | CAS | N | N | Y | N | NA | N | Attention difficulties | Slightly downslanting palpebral fissures, slight downturn of right corner of mouth | N |
| **40** | 6;2 | M | CAS | N | Y | N | N | NA | N | Emotional regulation difficulties | N | Asthma, eczema |
| **41** | 5;2 | M | CAS | N | Y | N | N | NA | N | Attention difficulties | N | N |
| **42** | 6;1 | M | CAS | Y | Y | N | N* | Corpus callosum mildly thick in places | N | ID, DCD | Small mouth, thin and slightly anteverted lips | Cleft palate (repaired) |
| **43** | 3;7 | M | Phonological disorder | Y | N | N | N | NA | N | N | N | N |
| **44** | 3;10 | F | CAS | N | N | N | N | NA | N | N | N | NA |
| **45** | 3;1 | M | CAS | N | Y | N | N* | N | N | N | N | N |
| **46** | 4;4 | M | CAS | N | Y | N | N | NA | N | N | N | Eczema, constipation |
| **47** | 3;11 | M | CAS, Phonological delay | N | N | N | N* | NA | N | N | N | N |
| **48** | 3;0 | M | CAS | N | N | N | N | NA | N | N | N | N |
| **49** | 4;9 | M | CAS | N | Y | N | N | NA | N | DCD | N | Bifid uvula |
| **50** | 3;11 | M | CAS | Y | Y | N | N | Normal | N | N | N | History of laryngomalacia, hypotonia, truncal ataxia |
| **51** | 3;2 | M | Features of CAS | N | N | N | N | NA | N | N | N | Sleep disturbances |
| **52** | 8;10 | M | CAS | Y | Y | N | N | A 4mm solitary focus of left frontal deep white matter T2 hyperintensity is nonspecific, and of uncertain clinical significance | Y | DCD | N | Multiple concussions, dental abscesses and caries, sleep difficulties, sensory difficulties, digestive issues, low heart rate and blood pressure, anxiety |
| **53** | 6;3 | M | CAS | Y | Y | N | N | NA | N | DCD | N | Coeliac disease |
| **54** | 7;8 | M | Phonological delay, features of CAS & dysarthria | Y | Y | Y | Y | N | N | DCD | N | Hypotonia |
| **55** | 4 | M | CAS, phonological disorder | Y | Y | N | N | NA | N | DCD | N | Hypoglycemia, undescended testes |
| **56** | 4;5 | M | CAS, phonological disorder | N | Y | N | N | NA | N | N | N | Undescended testes |
| **57** | 6;3 | M | CAS | N | N | N | N* | NA | N | N | N | Tonsillectomy, adenoidectomy, grommets |
| **58** | 5;7 | M | CAS | N | N | N | N | NA | N | N | N | Twin |
| **59** | 6;3 | F | CAS | Y | Y | N | N | N | N | DCD, GDD | N | Hypotonia |
| **60** | 5;4 | M | CAS, phonological delay | Y | Y | Y | N | NA | N | ASD | N | Asthma |
| **61** | 4;11 | F | Features of CAS, phonological disorder | N | N | N | N | NA | N | N | N | N |
| **62** | 7;3 | M | CAS | N | Y | N | N | NA | N | ASD features, GDD, attention difficulties | N | N |
| **63** | 4;10 | M | CAS, phonological disorder | Y | Y | Y | N* | NA | N | ASD | N | Grommets, tongue tie |
| **64** | 3;4 | M | CAS | Y | Y | N | N* | NA | N | N | N | Grommets, adenoidectomy, orbital dermoid |
| **65** | 7;8 | F | Dysarthria, features of CAS | Y | Y | N | N | NA | N | DCD | broad forehead, full cheeks, prominent upper central incisors | Adenoidectomy, tonsillectonmy, sleep disturbance, constipation |
| **66** | 3;7 | M | Dysarthria, phonological disorder | Y | Y | N | N | N | N | N | N | Hypoglycemia, hypotonia, moderate oropharyngeal dysphagia |
| **67** | 4;6 | M | Phonological disorder, features of CAS | Y | Y | N | N | N | N | GDD | Deep-set eyes, hanging columella, hypoplastic alae nasi, over-folded right helix and frontal bossing | Hypotonia, hypermobility, pulmonary valve developmental anomaly, trichorrhexis nodosa with photosensitivity |
| **68** | 3;7 | M | CAS | Y | Y | N | N* | NA | N | N | N | Grommets |
| **69** | 6;2 | F | CAS | N | N | N | N | NA | N | N | N | Nut allergy, periodic fever syndrome (resolved) |
| **70** | 4;4 | F | CAS | Y | Y | N | N* | NA | N | GDD, DCD | Micrognathia | Adenoidectomy, tonsillectonmy, grommets, increased femoral anteversion, asthma |

ASD, Autism spectrum disorder; CAS, Childhood apraxia of speech; DCD, Developmental coordination disorder; F, Female; GDD, Global developmental delay; ID, Intellectual disability; M, Male; N, Feature not present; NA, Not assessed; NDD, neurodevelopmental disorder; Y, Feature present; *Patient has a history of conductive hearing loss, treated with grommets, hearing was normal at the time of assessment.

Supplementary Table 1b: Extended linguistic phenotype and educational setting of patients without confirmed variants

| **Family** | Oral motor impairment | History of feeding issues | Language: receptive* | Language: expressive* | Reading deficits | Spelling deficits | Speech pathology | IQ | Education setting |
| --- | --- | --- | --- | --- | --- | --- | --- | --- | --- |
| **19** | Y | Y | Average | Mild | N | Y | Y | N (KBIT IQ Composite 110) | Mainstream |
| **20** | Y | NA | Average | Mild | NA | NA | Y | NA^ | Mainstream |
| **21** | N | Y | Average | Average | TY | TY | Y | NA^ | Not yet at school |
| **22** | Y | N | NA | NA | NA | NA | Y | NA^ | Mainstream |
| **23** | Y | Y | Y~ | Y~ | TY | TY | Y | NA^ | Not yet at school |
| **24** | Y | N | Severe | Severe | Y | Y | Y | Mild ID (FSIQ 65) | Mainstream |
| **25** | Y | N | Y~ | Y~ | Y | Y | Y | Moderate ID (FSIQ 41)^%^ | Mixed mainstream and specialist |
| **26** | Y | Y | Severe | Moderate | Y | Y | Y | NA^ | Specialist |
| **27** | N | N | Average | Mild | N | N | Y | NA^ | Mainstream |
| **28** | Y | N | Average | Average | TY | TY | Y | NA^ | Not yet at school |
| **29** | Y | Y | Severe | NA | Y | Y | Y | Mild-moderate ID (VCI 55-70, VSI 59-75, FSIQ 50-60) | Mainstream |
| **30** | N | Y | N~ | Y~ | TY | TY | Y | NA^ | Not yet at school |
| **31** | Y | Y | Average | NA | TY | TY | Y | NA^ | Not yet at school |
| **32** | Y | N | N~ | Y~ | Y | Y | Y | NA^ | Mainstream |
| **33** | Y | N | Severe | Severe | Y | Y | Y | N (PRI 99) | Mainstream |
| **34** | Y | N | Average | NA | Y | Y | Y | N (PRI 89) | Mainstream |
| **35** | Y | N | Mild | Y~ | Y | Y | Y | N (PRI 94) | Mainstream |
| **36** | Y | N | NS | NS | NA | NA | Y | NA^ | Specialist |
| **37** | Y | N | NA | NA | TY | TY | Y | NA^ | Not yet at school |
| **38** | Y | N | Average | Above average | TY | TY | Y | NA^ | Mainstream |
| **39** | Y | N | Mild | Mild | NA | NA | Y | N (PRI 112) | Mainstream |
| **40** | Y | N | Average | NA | NA | NA | Y | NA^ | Mainstream |
| **41** | N | Y | Average | Mild | NA | NA | Y | NA^ | Not yet at school |
| **42** | Y | Y | Mild | NA | Y | Y | Y | Mild-moderate ID (FSIQ 50-60) | Mainstream |
| **43** | Y | N | Mild | NS | TY | TY | Y | N (FSIQ 100) | Mainstream |
| **44** | Y | N | Average | Severe | TY | TY | Y | NA^ | Mainstream |
| **45** | Y | N | Average | NA | TY | TY | Y | NA^ | Not yet at school |
| **46** | Y | N | Above average | Average | TY | TY | Y | NA^ | Mainstream |
| **47** | Y | Y | Above average | NA | TY | TY | Y | NA^ | Mainstream |
| **48** | Y | N | NA | NA | TY | TY | Y | NA^ | Mainstream |
| **49** | Y | Y | Average | Average | TY | TY | Y | NA^ | Mainstream |
| **50** | Y | Y | Severe | NS | TY | TY | Y | N (PRI average) | Mainstream |
| **51** | Y | Y | Average | NS | TY | TY | Y | N (FSIQ 93) | Mainstream |
| **52** | Y | N | Average | Average | N | N | Y | N (FSIQ average) | Mainstream |
| **53** | Y | Y | Moderate | Mild | Y | Y | Y | NA | Mainstream |
| **54** | Y | Y | Average | Severe | Y | Y | Y | Borderline (FSIQ 76) | Mainstream |
| **55** | Y | N | Average | Average | TY | TY | Y | N (KBIT non-verbal IQ 99) | Mainstream |
| **56** | Y | Y | Above average | Average | TY | TY | Y | NA^ | Mainstream |
| **57** | N | N | Average | NA | N | N | Y | N (PRI 101) | Mainstream |
| **58** | Y | N | Average | Severe | N | N | Y | N (PRI 121) | Mainstream |
| **59** | Y | Y | Average | Average | N | N | Y | N (KBIT IQ Composite 105) | Mainstream |
| **60** | N | Y | Severe | Severe | N | N | Y | Borderline (KBIT IQ Composite 72) | Mainstream |
| **61** | N | N | NA | NA | TY | TY | Y | NA^ | Mainstream |
| **62** | Y | NA | Severe | Severe | NA | NA | Y | N (non-verbal IQ average) | NA |
| **63** | Y | N | Severe | Severe | TY | TY | Y | NA^ | Mainstream |
| **64** | Y | N | N~ | NS | TY | TY | Y | NA^ | Mainstream |
| **65** | Y | N | Severe | Severe | Y | Y | Y | N (FSIQ 105) | Mainstream |
| **66** | NS | Y | Average | Average | TY | TY | Y | NA^ | Mainstream |
| **67** | Y | N | NA | NA | TY | TY | Y | NA^ | Specialist |
| **68** | Y | Y | Severe | Severe | TY | TY | Y | NA^ | Not yet at school |
| **69** | Y | N | Average | Severe | N | N | Y | N (FSIQ 101) | Mainstream |
| **70** | Y | NA | Severe | Severe | TY | TY | Y | Mild (VCI 77, FSIQ 69) | Not yet at school |

FSIQ, Full scale IQ; N, no; NA, Not assessed; NS, unable to score assessment, abilities likely severe range; N~, based on subtest scaled scores; VCI: Verbal Comprehension Index; VSI: Visual Spatial Comprehension; PRI, Perceptual reasoning index; TY; not applicable, too young (< 5 years old) to assess literacy; Y, feature present.

*Language severity rated according to CELF-5 as follows: 86-114 average, 78-85 mild, 71-77 moderate, <70 severe.

^ Assessment not indicated by the family or treating physician; % Participant received ID diagnosis after recruitment.
